# Accuracy of Genomic Evaluations of Juvenile Growth Rate in Common Carp (*Cyprinus carpio*) Using Genotyping by Sequencing

**DOI:** 10.3389/fgene.2018.00082

**Published:** 2018-03-13

**Authors:** Christos Palaiokostas, Martin Kocour, Martin Prchal, Ross D. Houston

**Affiliations:** ^1^The Roslin Institute, Royal (Dick) School of Veterinary Studies, University of Edinburgh, Edinburgh, United Kingdom; ^2^Faculty of Fisheries and Protection of Waters, South Bohemian Research Centre of Aquaculture and Biodiversity of Hydrocenoses, University of South Bohemia in České Budějovice, Vodňany, Czechia

**Keywords:** aquaculture breeding, carps, high-throughput sequencing, RAD-seq, genomic prediction

## Abstract

Cyprinids are the most important group of farmed fish globally in terms of production volume, with common carp (*Cyprinus carpio*) being one of the most valuable species of the group. The use of modern selective breeding methods in carp is at a formative stage, implying a large scope for genetic improvement of key production traits. In the current study, a population of 1,425 carp juveniles, originating from a partial factorial cross between 40 sires and 20 dams, was used for investigating the potential of genomic selection (GS) for juvenile growth, an exemplar polygenic production trait. RAD sequencing was used to identify and genotype SNP markers for subsequent parentage assignment, construction of a medium density genetic map (12,311 SNPs), genome-wide association study (GWAS), and testing of GS. A moderate heritability was estimated for body length of carp at 120 days (as a proxy of juvenile growth) of 0.33 (s.e. 0.05). No genome-wide significant QTL was identified using a single marker GWAS approach. Genomic prediction of breeding values outperformed pedigree-based prediction, resulting in 18% improvement in prediction accuracy. The impact of reduced SNP densities on prediction accuracy was tested by varying minor allele frequency (MAF) thresholds, with no drop in prediction accuracy until the MAF threshold is set <0.3 (2,744 SNPs). These results point to the potential for GS to improve economically important traits in common carp breeding programs.

## Introduction

Carps have the highest global production volume of all aquaculture fish (FAO, 2015) and are farmed in a wide variety of environments and production systems (Balon, 1995). In common with most aquaculture species, only a minority of farmed common carp are derived from family-based selective breeding programs, and crossbreeding of partially inbred strains is commonly applied to benefit from heterosis (Hulata, 1995; Vandeputte, 2003; Janssen et al., 2017). Family-based programs have the advantage of enabling cumulative increases in economic traits, and maintaining a high degree of control of the level of inbreeding of stocks. Empirical data relating to selective breeding of family-based programs in several fish species show an increase in genetic gain of up to 15% per generation (Gjedrem, 2000). While initial studies suggested that within breed selection is ineffective in common carp (Moav and Wohlfarth, 1976), recent studies focusing on growth and survival traits highlighted the potential of applying selective breeding to enhance production (Kocour et al., 2007; Vandeputte et al., 2008; Nielsen et al., 2010; Ninh et al., 2013; Dong et al., 2015).

Traditional pedigree-based selective breeding incorporating best linear unbiased predictor (BLUP) methodology (Henderson, 1975) has greatly benefited both animal and plant agriculture. Nevertheless, the utilization of just the between-family component of genetic variation imposes limitations to selection accuracy and therefore genetic gain (Meuwissen et al., 2013). Selective breeding can be significantly enhanced by the application of genomic tools, via improvement of selection accuracy and potentially also by identification of causative factors impacting on key production traits (Meuwissen et al., 2001, 2013; Hickey et al., 2017). Genomic selection (GS) involves the simultaneous use of genome-wide genetic markers to estimate breeding values for selection candidates utilizing both within and across family variation (Meuwissen et al., 2001). By using all markers in the calculation of breeding values, GS overcomes the limitations of marker-assisted selection for such traits, where typically only a small percentage of genetic variation can be utilized for polygenic traits (Daetwyler et al., 2013). In aquaculture species, GS has been enabled by the increased technical feasibility and reduced cost of generation of genome-wide marker data in non-model organisms via SNP arrays or genotyping by sequencing (Davey et al., 2011; Robledo et al., 2017).

The effectiveness of GS at deriving more accurate breeding values than traditional pedigree-based selection has been demonstrated using simulated and empirical data in both livestock and aquaculture (Goddard and Hayes, 2009; Sonesson and Meuwissen, 2009). Empirical data collected to date suggest that the majority of traits of interest for animal production (e.g., growth and disease resistance) are underpinned by a polygenic genetic architecture (de los Campos et al., 2013). In aquaculture species, where large full-sibling family sizes are typically available, the advantages of genomic prediction of breeding values in aquaculture species are clear from several studies of such polygenic traits (e.g., Odegård et al., 2014; Tsai et al., 2015, 2016; Vallejo et al., 2017), albeit genomic prediction is less effective when only distant relatives are used in deriving the prediction equation (Tsai et al., 2016).

Restriction-site-associated DNA sequencing (RAD-seq) is a reduced representation of high-throughput sequencing technique for the concurrent detection and genotyping of SNP markers (Baird et al., 2008). RAD-seq and similar genotyping by sequencing techniques rely on digestion of the genomic DNA with a restriction enzyme, and subsequent high-depth sequencing of the flanking regions. These techniques have been widely applied due to their cost-efficiency in a wide range of aquaculture species (Robledo et al., 2017), both in genome-wide association studies (GWAS) (e.g., Campbell et al., 2014; Palti et al., 2015; Barria et al., 2017) and GS studies (e.g., Palaiokostas et al., 2016; Vallejo et al., 2016). The main aim of this study was to investigate the potential of genomic prediction of an exemplar polygenic trait in common carp (juvenile growth) using genome-wide SNP markers generated by RAD-seq. To achieve this, samples of 1,425 carp measured for body weight and length at approximately 4 months of age were used. RAD-seq was used to genotype genome-wide SNP markers, parentage assignment was performed, and heritability (of body weight and length) was estimated. The obtained SNPs were utilized for construction of a medium density linkage map, followed by a GWAS to test the association between individual loci and growth. Finally, GS was tested to evaluate the potential of incorporating genomic data for selective breeding compared to pedigree-based selection using this exemplar polygenic trait.

## Materials and Methods

### Sample Collection

A population of Amur Mirror Carp was created at the University of South Bohemia, Czech Republic in May 2014 using artificial insemination (Vandeputte et al., 2004) involving four factorial crosses each comprising 5 dams × 10 sires (20 dams and 40 sires in total). Incubation of eggs was performed in 9 lt Zugar jars at 20°C. At the swimming stage, randomly sampled progeny of the same total volume from each mating was pooled and reared under semi-intensive pond conditions throughout the growing season (from May to September). In September, a sample of 1,425 fish was fin-clipped, passive integrated transponder (PIT)-tagged, weighed to the nearest 0.01 g, and measured for standard length (SL) (from the tip of the nose to the end of the caudal peduncle) to the nearest millimeter. All working procedures complied with the European Union Directive 2010/63/EU for the protection and welfare of animals used for scientific purposes.

### RAD Library Preparation and Sequencing

Genomic DNA was extracted from fin samples using the REALPure genomic DNA extraction kit (Durviz S.L.) and treated with RNase. Each sample was quantified by spectrophotometry (Nanodrop), and its quality was assessed by agarose gel electrophoresis, before being diluted to a concentration of 20 ng/μL [measured by Qubit Fluorometer (Invitrogen)] in 5 mmol/L Tris, pH 8.5.

The RAD-specific P1 and P2 paired-end adapters and library amplification PCR primer sequences used in this study are detailed in Baxter et al. (2011). Briefly, each sample (0.72 μg parental DNA/0.24 μg offspring DNA) was digested at 37°C for 60 min with *Sbf*I (recognizing the CCTGCA| GG motif) high fidelity restriction enzyme (New England Biolabs, NEB). The reactions (12 μL final volumes) were then heat inactivated at 65°C for 20 min. Individual-specific P1 adapters, each with a unique 5 bp barcode, were ligated to the *Sbf*I-digested DNA at 20°C for 60 min by adding 1.8/0.6 μL 100 nmol/L P1 adapter, 0.45/0.15 μL 100 mmol/L rATP (Promega), 0.75/0.25 μL 10× Reaction Buffer 2 (NEB), 0.36/0.12 μL T4 ligase (NEB, 2 M U/mL), and reaction volumes made up to 45/15 μL with nuclease-free water for each parental/offspring sample. Following heat inactivation at 65°C for 20 min, the ligation reactions were slowly cooled to room temperature (over 1 h) then combined in appropriate multiplex pools. Shearing and initial size selection (300–600 bp) by agarose gel separation were followed by gel purification, end repair, dA overhang addition, P2 (individual-specific adapters) paired-end adapter ligation, library amplification, as described in the original RAD protocol (Baird et al., 2008; Etter et al., 2011). A total of 150 μL of each amplified library (14–17 PCR cycles, library dependent) was size selected (ca. 400–700 bp) by gel electrophoresis as described in Houston et al. (2012). Following a final gel elution step into 20 μL EB buffer (MinElute Gel Purification Kit, Qiagen), 66 libraries (24 animals each) were sent to BMR Genomics (Italy), for quality control and high-throughput sequencing. Libraries were run in 14 lanes of an Illumina NextSeq 500, using 75 base paired-end reads (v2 chemistry). The sequence reads were deposited at the NCBI Sequence Read Archive (SRA) under the accession PRJNA414021.

### Genotyping RAD Alleles – SNP Identification

Reads missing the restriction site, with ambiguous barcodes and PCR duplicates, were identified and discarded using the Stacks software 1.4 (Catchen et al., 2011). The remaining reads were aligned to the common carp reference genome assembly version *GCA_000951615.2* (Xu et al., 2014) using bowtie2 (Langmead and Salzberg, 2012). The aligned reads were sorted into loci and genotypes using the Stacks software 1.4 (Catchen et al., 2011). A minimum stack depth of at least 10 or 5 was required for the parental and offspring samples, respectively. Only loci containing one or two SNPs were considered for downstream analysis. SNPs with minor allele frequency (MAF) <0.01, >25% missing data, and deviating from expected Hardy–Weinberg equilibrium in the parental samples (*P* < 1e-06) were discarded.

### Parentage Assignment

Due to the partial factorial crosses performed in this experiment, the family structure of the offspring was unknown at the time of sampling. Parentage assignment was performed using the RAD-seq-derived SNP data and the R/hsphase (Ferdosi et al., 2014) software allowing for a maximum overall genotyping error of 4%. The pedigree obtained was further validated for possible erroneous assignments using FImpute (Sargolzaei et al., 2014).

### Linkage Map Construction

Linkage map construction was performed using Lep-Map v2 (Rastas et al., 2013). SNPs with MAF <0.05 in individual families and those deviating from expected Mendelian segregation (*P* < 0.001) were excluded. Linkage groups were formed using a minimum LOD threshold value of 18 in the “*SeparateChromosomes*” module, allowing a maximum distance between consecutive SNPs of 50 cM. Marker order within each linkage group was performed using the “*OrderMarkers*” module using the *SexAverage* option. Map distances were calculated in centiMorgans (cM) using the Kosambi mapping function.

### Heritability Estimation

Heritability estimates of weight and length were performed using both the pedigree-based relationship matrix and the genomic relationship matrix. Variance components was estimated using AIREMLF90 (Misztal et al., 2002) with the following animal model:

(1) y=Xb+Zu+e,

where y is the vector of recorded phenotypes, b vector of the fixed effects (the four-level factorial cross), X the incidence matrix relating phenotypes with the fixed effects, Z the incidence matrix relating phenotypes with the random animal effects, u the vector of random animal effects ∼*N*(0, A${\text{σ}}_{\text{g}}^{2}$) with A corresponding to the pedigree-based relationship matrix or the genomic relationship matrix G (VanRaden, 2008), ${\text{σ}}_{\text{g}}^{2}$ the additive genetic variance, e the vector of residuals ∼*N*(0, Iσ_e_^2^) and ${\text{σ}}_{\text{e}}^{2}$ the residual variance.

Heritability was estimated using the following formula:

$$h^{2}=\frac{{\text{σ}}_{\text{g}}^{2}}{{\text{σ}}_{\text{g}}^{2}+{\text{σ}}_{\text{e}}^{2}}.$$

Bivariate models with the same fixed and random effects as in Equation (1) were used in order to estimate genetic correlations between the phenotypes of weight and length.

### Genome-Wide Association Analysis

To test the association between individual SNPs and growth (only length records were utilized), GWAS was performed using R/gaston (Hervé and Dandine-Roulland, 2018). The mixed model applied had the same format as in Equation (1) with the addition of each tested SNP as a fixed effect. The genome-wide significance threshold was calculated using a Bonferroni correction (0.05/*N*), where *N* represents the number of QC-filtered SNPs.

### Genomic Selection

The accuracy of prediction of genomic breeding values (GEBVs) was calculated and benchmarked against the accuracy of prediction for EBVs using traditional pedigree-based best linear unbiased prediction (BLUP) (Henderson, 1975). GEBVs were estimated with GBLUP (Meuwissen et al., 2001) using the BLUPF90 suite (Misztal et al., 2002) updated for genomic analyses (Aguilar et al., 2011). Pedigree-based BLUP was applied to calculate breeding values using the same software. The general form of the fitted models was as in Equation (1).

A fivefold cross-validation was performed in order to test prediction accuracy. The data set was randomly split into sequential non-overlapping training (*n* = 972 individuals) and validation sets (*n* = 242). The fivefold cross-validation procedure was repeated 10 times in order to reduce random sampling effects. The accuracy of the estimated breeding values was approximated as:

r=(EBV,y)/h,

where y is the vector of recorded phenotypes and *h* the square root of the heritability. GEBVs were used to approximate accuracy in the case of GBLUP. Additionally, five different scenarios were used in order to test the effect of reduced genotyping densities on the obtained prediction accuracies. In these scenarios, GBLUP was performed as above using subsets of SNPs selected by progressive increase of a minimum threshold for MAF. The tested scenarios involved MAF thresholds of 0.1 (8,237 SNPs), 0.2 (4,950 SNPs), 0.3 (2,744 SNPs), 0.4 (1,182 SNPs), and 0.45 (530 SNPs). Bias in the form of the regression coefficient of the phenotypic trait on (G)EBV was estimated for both PBLUP and all tested scenarios of GBLUP.

## Results

### Descriptive Statistics

**FIGURE 1:**
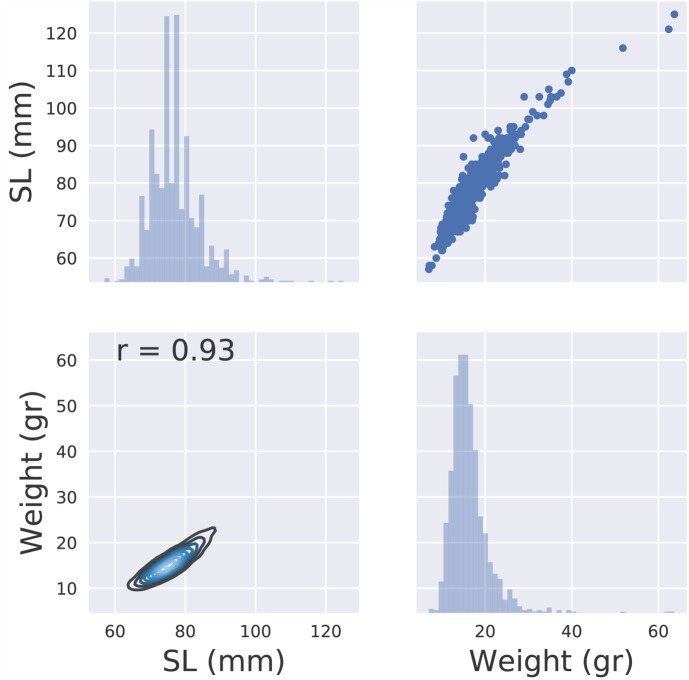
Descriptive statistics of the phenotypic data (weight, length).

The mean weight of the genotyped carp juveniles after approximately 4 months of growth (**Supplementary Table S1**) was 16.3 g (SD 4.6) and the mean SL was 77 mm (SD 7.1). The Pearson correlation coefficient between length and weight was *r* = 0.93 (**Figure 1**).

### SNP Identification and Genotyping

Animals with fewer than 25% missing SNP genotypes were retained for downstream analysis (corresponding to 60 parental and 1,400 offspring samples). The total number of raw reads passing the QC filters was 6.89 (SD 1.33) M for the parental samples and 3.68 (SD 1.28) M for the offspring. The mean number of RAD loci identified was 57,983 (SD 1,573) and 57,235 (SD 5,224) for parents and offspring, respectively, with mean coverage of 60 (SD 10) X and 29 (SD 8) X, respectively. A total of 22,756 putative SNPs were identified, of which 20,039 SNPs passed QC filters and were retained for downstream analysis (**Supplementary Table S2**).

### Parentage Assignment

The carp progeny was assigned to unique parental pairs allowing for a maximum genotypic error rate of 4%. In total 1,214 offspring were uniquely assigned, forming 195 full-sib families (40 sires, 20 dams) ranging from 1 to 21 animals per family with a mean size of 6 (SD 4). The individual dam contribution to the population ranged from 9 to 99 animals with a mean of 61 (SD 23), while the sire contribution ranged from 7 to 53 animals with a mean of 30 (SD 12).

### Linkage Map Construction

The linkage map constructed using the aforementioned families consisted of 12,311 SNPs (**Table 1**) that were grouped into 50 linkage groups (in accordance with the expected karyotype). The length of the consensus linkage map was 3,944 cM (**Supplementary Table S3**). The number of SNPs per chromosome ranged from 157 to 350 (mean = 246; SD = 45), while linkage group length ranged between 67 and 106 cM (mean = 79; SD = 9).

**Table 1:** Number of QC-filtered SNPs per linkage group.

Linkage group	Size (cM)	Number of markers
1	102	350
2	91	350
3	76	325
4	77	322
5	90	319
6	77	315
7	77	311
8	77	292
9	75	290
10	84	287
11	83	283
12	71	277
13	81	272
14	70	263
15	69	260
16	78	259
17	105	257
18	78	256
19	92	249
20	75	249
21	106	246
22	75	245
23	84	241
24	70	241
25	76	240
26	77	239
27	71	238
28	84	237
29	93	237
30	81	236
31	71	229
32	77	229
33	78	227
34	70	227
35	87	226
36	77	225
37	88	222
38	77	221
39	73	215
40	71	213
41	74	212
42	72	212
43	72	208
44	79	203
45	80	199
46	67	193
47	73	175
48	71	169
49	75	163
50	69	157
Total	3,944	12,311

### Heritability Estimation

The estimated heritabilities for weight and length were 0.26 (SE 0.05) and 0.33 (SE 0.05), respectively, and were consistent between the pedigree and genomic models. Genetic correlation between body weight and SL was 0.94 (SE 0.02), and as such only length data were used for downstream analyses.

### Genome-Wide Association Study (GWAS) – Genomic Selection (GS)

**FIGURE 2:**
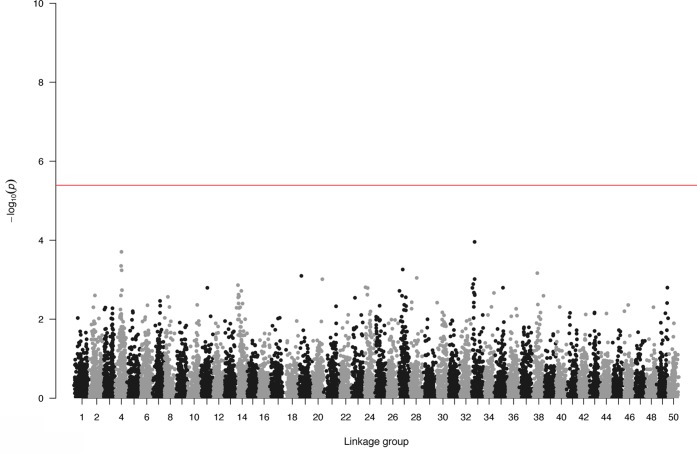
Genome-wide association plot for body length.

**FIGURE 3:**
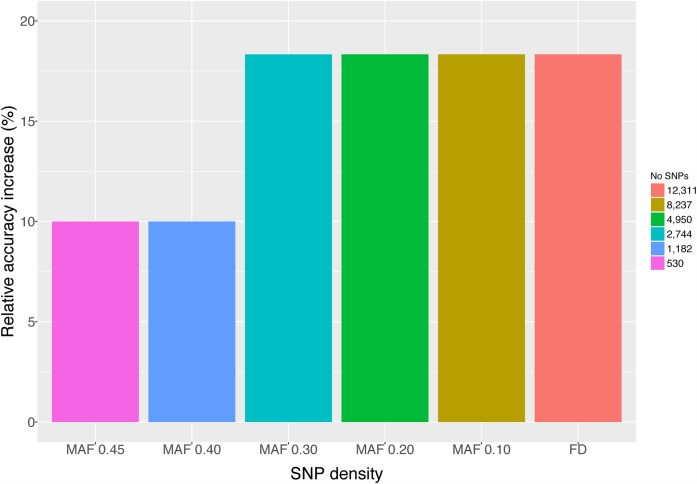
Mean accuracies of GBLUP and PBLUP. GBLUP accuracies were obtained using either all available SNPs (FD; 12,311), SNPs with MAF >0.10 (8,237), SNPs with MAF >0.20 (4,950), SNPs with MAF >0.30 (2,744), SNPs with MAF >0.40 (1,182), and SNPs with MAF >0.45 (530).

Genome-wide association study and GS were performed using only the SL data. GWAS did not identify SNPs surpassing the genome-wide significant threshold (**Figure 2**), indicating that juvenile growth is likely to be a polygenic trait. Using the cross-validation approach, breeding value prediction accuracy was estimated to be 0.60 (SE 0.03) for PBLUP, as opposed to 0.71 (SE 0.03) with GBLUP. Accuracies obtained through GBLUP using the various reduced SNP densities ranged from 0.66 to 0.71 (**Figure 3**). GBLUP using only SNPs with a minimum MAF of 0.45 (530 SNPs) had approximately a 10% accuracy improvement compared to PBLUP. SNP densities of a minimum MAF of 0.3 had practically the same prediction accuracies as the full data set (**Figure 3**). Estimated bias of (G)EBVs ranged between 0.78 and 1.02. The scenario using SNPs with minimum MAF of 0.45 was found to produce the most biased GEBVs (**Table 2**).

**Table 2:** Mean accuracy of pedigree BLUP (PBLUP) and GBLUP using SNP of varying MAF obtained from 10 repeats of fivefold cross-validation.

Reps	FD^1^ (bias)	MAF^2^ > 0.1 (bias)	MAF^3^ > 0.2 (bias)	MAF^4^ > 0.3 (bias)	MAF^5^ > 0.4 (bias)	MAF^6^ > 0.45 (bias)	PBLUP (bias)
1st	0.71 (1.05)	0.71 (1.04)	0.71 (1.05)	0.71 (1.06)	0.68 (1.03)	0.66 (0.93)	0.59 (1.02)
2nd	0.70 (1.03)	0.71 (1.02)	0.70 (1.02)	0.70 (1.03)	0.66 (0.99)	0.65 (0.90)	0.59 (1.01)
3rd	0.73 (1.05)	0.73 (1.04)	0.73 (1.05)	0.73 (1.05)	0.67 (0.99)	0.68 (0.92)	0.61 (1.03)
4th	0.69 (0.98)	0.69 (0.97)	0.69 (0.99)	0.69 (0.99)	0.64 (0.95)	0.63 (0.84)	0.60 (1.02)
5th	0.69 (1.04)	0.68 (1.00)	0.70 (0.99)	0.69 (1.02)	0.66 (0.98)	0.66 (0.91)	0.58 (1.02)
6th	0.71 (1.03)	0.71 (1.02)	0.71 (1.03)	0.71 (1.03)	0.67 (1.00)	0.66 (0.90)	0.61 (1.05)
7th	0.70 (1.03)	0.70 (1.03)	0.70 (1.03)	0.70 (1.02)	0.65 (0.97)	0.65 (0.87)	0.60 (1.02)
8th	0.71 (1.04)	0.71 (1.04)	0.71 (1.04)	0.71 (1.05)	0.66 (1.00)	0.66 (0.92)	0.60 (1.03)
9th	0.71 (1.01)	0.71 (1.02)	0.71 (1.03)	0.71 (1.04)	0.67 (1.00)	0.67 (0.92)	0.61 (1.04)
10th	0.70 (1.04)	0.70 (1.02)	0.70 (1.03)	0.70 (1.03)	0.65 (0.98)	0.65 (0.88)	0.60 (1.02)
Mean	0.71 (1.03)	0.71 (1.02)	0.71 (0.98)	0.71 (0.87)	0.66 (0.99)	0.66 (0.90)	0.60 (1.03)

*^1^12,311 SNPs, ^2^8,237 SNPs, ^3^4,950 SNPs, ^4^2,744 SNPs, ^5^1,182 SNPs, ^6^530 SNPs.*

## Discussion

Traditional selective breeding relies on well-documented pedigree, which is relatively straightforward in livestock, but more challenging in aquaculture species. Genetic markers have the potential of addressing this issue, facilitating the practical implementation of breeding programs via effective parentage assignment and circumventing the requirement of rearing the fish in separate tanks until tagging is possible (Vandeputte and Haffray, 2014). In the current study, the utility of RAD-seq data for enabling selective breeding for a polygenic trait in common carp was investigated. Using the RAD SNP data, approximately 86% of genotyped animals could be uniquely assigned to parental pairs. Following pedigree reconstruction, moderate heritability estimates of 0.26 and 0.33 were obtained for juvenile weight and length, respectively. These estimates are in line with the previous heritability estimates of weight/length obtained from juvenile carp (Vandeputte et al., 2004; Ninh et al., 2013; Hu et al., 2017). However, one limitation of the current study lies in the fact that trait measurements were taken in juveniles, and the correlation between growth in early life with growth to harvest size is unknown for this population. Contradicting evidence is available regarding this, with studies recording high positive phenotypic correlations between growth-related traits at juvenile and harvest stage (Ninh et al., 2013), moderate positive correlations (Vandeputte et al., 2008; Nielsen et al., 2010), or correlations near to zero (Hu et al., 2017). However, common carp juvenile weight and length in the current study is used as an exemplar polygenic trait, with broader implications for the use of genomic data to improve other economically important traits, in particular for those typically not measurable directly on selection candidates (disease resistance or fillet traits, e.g.).

Genetic markers can be a valuable addition to selective breeding, but the optimal strategy for their application depends on the underlying genetic architecture of the trait of interest. Where a trait is primarily controlled by one or several major QTL, it may be most effective to use marker-assisted selection with low-density markers flanking QTL regions. This is the case for resistance to the Infectious Pancreatic Necrosis virus in Atlantic salmon for example, where almost all genetic variation is explained by a single QTL (Houston et al., 2008; Moen et al., 2009). However, most traits of economic importance have a polygenic architecture and GS is likely to be the most effective use of genetic markers to improve these traits. In the current study, the GWAS results implied that the juvenile growth traits were polygenic in nature. Previous studies using linkage analysis have reported QTL related to growth in common carp. A study on a single full-sibling family of common carp detected 14 QTL distributed in five different LGs, including regions associated with the hypothalamic–pituitary–gonadal and GH/IGF-I axis that regulates development, cell-proliferation, energy metabolism, and growth (Peng et al., 2016). Additionally, a study on eight full-sib families of common carp reported 38 growth-related QTL, although no QTL was detected in all of the families (Lv et al., 2016). Therefore, due to the lack of consistent major effect QTL, it is unlikely that MAS will be an effective approach for selecting the best breeding candidates for this and similar economically important traits, and GS is a promising alternative.

The results from the testing of GS in the carp population used in the current study were encouraging, with prediction accuracy obtained through cross-validation analyses using GBLUP being 0.71. This signifies an approximate 18% improvement of accuracy compared to pedigree BLUP, suggesting major potential benefits for selection accuracy and genetic gain for complex economic traits in carp. Our results are in agreement with other studies in aquaculture species where a major benefit to using the genomic models was demonstrated for disease resistance in, e.g., Atlantic salmon (Tsai et al., 2015, 2016), rainbow trout (Vallejo et al., 2017), and gilthead sea bream (Palaiokostas et al., 2016). GS benefits from increased sample size of the reference population (Vallejo et al., 2017) indicating that further improvements of prediction accuracies could be expected by increasing the number of genotyped animals in the current study. In typical aquaculture breeding designs, including mass spawning species, where the reference and validation sets are closely related use of genetic markers can be highly effective for capturing within-family genetic variation (Lillehammer et al., 2013). However, given that prediction accuracy is likely to be highly reliant on genetic relationships, this implies that genomic prediction in distant relatives to the reference population is likely to be substantially more challenging, as observed in terrestrial livestock (Daetwyler et al., 2013).

It is noteworthy that GBLUP resulted in increased prediction accuracies compared to pedigree prediction using relatively sparse (∼500 SNPs) genotype data, especially considering the large genome size (∼1.8 Gb) of common carp. Similar results were recorded for Atlantic salmon where similar prediction accuracies were obtained from 5K SNPs as for 112K SNPs (Tsai et al., 2015). Both common carp and Atlantic salmon have large genomes, and the effectiveness of genomic prediction at low marker density is again likely to reflect the aforementioned close relationships between the training and validation animals. Nonetheless, this may have economic benefits, since low cost sparse genotyping could be sufficient for improving prediction accuracies in a breeding program. This could be important for driving implementation of GS, since breeding candidates of aquaculture species are of lower economic value compared with livestock, making the application of costly high-density genotyping approaches difficult to justify. Genotype imputation approaches have major potential to drive this genotype density and cost down further, and have already shown significant promise in Atlantic salmon (Tsai et al., 2017). While verification of the results of the current study using harvest size carp (or other economically important complex traits) would be a logical next step, the results of the current study suggest that GS has potential for substantial improvement in prediction accuracy in carp breeding, and RAD-seq is one method of generating the marker data to enable this improvement.

## Conclusion

The results from the current study demonstrated that the use of SNP markers generated via RAD-seq is an efficient approach for investigating and potentially improving a polygenic trait in a common carp breeding population. These SNPs enabled pedigree assignment, genetic parameter estimation, GWAS, and GS within a single experiment. GS resulted in improved prediction accuracy versus pedigree approaches even when only relatively sparse marker information was utilized.

## Author Contributions

MK, MP, and RH conceived the study and contributed to designing the experimental structure. MK and MP shared on establishing and on-growing the experimental stock, PIT tagging, phenotype data recording, and fin clipping the fish. CP carried out the DNA extractions, RAD library preparation, and sequence data processing. CP and RH carried out parentage assignment and the quantitative genetic analyses. All authors contributed to drafting the manuscript.

## Conflict of Interest Statement

The authors declare that the research was conducted in the absence of any commercial or financial relationships that could be construed as a potential conflict of interest.

## Supplementary Material

TABLE S1 Parentage and phenotypic information of juvenile common carp.

TABLE S2 Detailed information of identified SNP through RADseq.

TABLE S3 Linkage map of common carp.
